# The first German map of georeferenced ixodid tick locations

**DOI:** 10.1186/s13071-014-0477-7

**Published:** 2014-10-10

**Authors:** Franz Rubel, Katharina Brugger, Masyar Monazahian, Birgit Habedank, Hans Dautel, Sandra Leverenz, Olaf Kahl

**Affiliations:** Institute for Veterinary Public Health, University of Veterinary Medicine Vienna, Veterinärplatz 1, A-1210 Vienna, Austria; Governmental Institute of Public Health of Lower Saxony, Hannover, Germany; Section IV 1.4 - Public Health Pest and their Control, Federal Environment Agency, Berlin, Germany; tick-radar GmbH, Berlin, Germany

**Keywords:** *Ixodid ticks*, *Ixodes*, *Dermacentor*, *Haemaphysalis*, *Hyalomma*, Distribution map, Dataset

## Abstract

**Background:**

Georeferenced locations of ixodid ticks are required to depict the observed distribution of species. Further, they are used as input data for species distribution models also known as niche models. The latter were applied to describe current and future (projected) tick distributions. Beside model assumptions and selected climate parameters, the number of georeferenced tick locations available as a digital dataset is of fundamental importance for the reliability of such models. For Germany, however, no comprehensive dataset of ixodid tick species exists. The goal of this study was to put together all the available information on ixodid tick locations in Germany to produce such a digital dataset and to visualize it in a map.

**Findings:**

A total of 2,044 georeferenced locations of ixodid ticks in Germany were compiled from two existing datasets (altogether 993 locations) and an extensive literature study (1,051 locations). The resulting digital dataset comprises the following tick species: *Ixodes ricinus* (1,855 locations), *Ixodes apronophorus* (1), *Ixodes frontalis* (1), *Ixodes hexagonus* (1), *Ixodes trianguliceps* (4), *Dermacentor marginatus* (77), *Dermacentor reticulatus* (96), *Haemaphysalis concinna* (8) and *Hyalomma marginatum* (1). The data were used to draw a tick map for Germany, showing *I. ricinus* occurring in the whole federal territory, while *D. marginatus* has been restricted to the climatically favoured region of the Rhine valley. Clustered locations of *D. reticulatus* were also documented in the Rhine valley as well as in Berlin and its vicinity.

**Conclusions:**

The introduced map depicts for the first time the available geographical coordinates of ixodid tick locations in Germany. The digital dataset used to draw the map is provided to the scientific community as a basis for further investigations such as species distribution modelling.

**Electronic supplementary material:**

The online version of this article (doi:10.1186/s13071-014-0477-7) contains supplementary material, which is available to authorized users.

## Background

Tick species distribution changes according to climate, land use patterns and forest management [1]. Therefore, it is of particular interest to develop scenarios on future distributions of ticks and tick-borne diseases based on climate models and the available knowledge of tick ecology and physiology. Among other methods, species distribution models reconstructing the climatic niche were applied. They describe the potential distribution of a given species, which may be projected into the future by using climate model predictions. For example, the current and future distribution of *Ixodes ricinus* in Europe was investigated [2], using the geographical coordinates of *I. ricinus* locations from another study [3]. Beside model assumptions and selected climate parameters, the number of georeferenced tick locations available as a digital dataset is of fundamental importance for the reliability of modelling results. They comprise distributions of tick species as well as the distribution patterns of tick-borne diseases such as tick-borne encephalitis, Lyme borreliosis [4], babesiosis [5,6], rickettsiosis [7] and others [8].

Digital datasets covering Europe were provided by Estrada-Peña et al. [3] and GBIF, the Global Biodiversity Information Facility [9]. Both datasets feature major data gaps especially for Germany. The same is true for Belgium, where a recently published study on the distribution of ixodid tick species [10] is not included in those two datasets so far. The provision of exact geographical coordinates of all the described locations of ticks and tick-borne pathogens should be standard in all modern papers. Georeferences may be included in digital datasets and used in subsequent studies, especially in those investigating the spatio-temporal distributions of ticks on a continental scale. For Germany, however, such comprehensive data as presented for Belgium [10] are missing so far. To reduce these shortcomings a literature study on georeferenced tick locations was performed. Results comprise of coordinates extracted from recent papers, extracted from restricted papers mainly published in German language, digitized from historical hand-drawn maps and added from unpublished material, i.e. kindly provided by colleagues.

Digitized locations, of course, are of lower accuracy than locations described by geographical coordinates determined by GPS in the field. Thus, accuracy measures were given for all data referenced in Table 1. It is distinguished between high (h), medium (m), low (l) and unknown (u) accuracy. A high accuracy (±30 m) was allocated to coordinates given in degrees, minutes and seconds or in decimal degrees with at least 4–5 relevant decimal places. A medium accuracy (±1 km) was assumed for coordinates given in degrees and minutes or in decimal degrees with at least 2–3 relevant decimal places. A medium accuracy was also assumed for ticks collected from animals (e.g. deer, dogs) or humans and for coordinates digitized from local maps. Coordinates digitized from regional maps were classified as low-accuracy data (±10 km).

**Table 1:** **Number of georeferenced ixodid tick sampling sites in Germany compiled in this study**

**No. Sites**	**Species**	**Accuracy**	**References**
661	*I. ricinus*	h	MM, OK
1	*I. ricinus*	h	[4]
13	*I. ricinus*	h	[11]
217	*I. ricinus*	u	[9,12]
8	*I. ricinus*	h	[13]
10	*I. ricinus*	h	[14]
776	*I. ricinus*	u	[3]
6	*I. ricinus*	h	[15]
6	*I. ricinus*	h	[16,17]
20	*I. ricinus*	h	[18]
9	*I. ricinus*	h	[19]
5	*I. ricinus*	h	[20]
58	*I. ricinus*	l	[21]
64	*I. ricinus*	l	[22]
1	*I. ricinus*	m	[23]
1	I.apronophorus	m	[23]
1	*I. frontalis*	h	[24]
1	*I. hexagonus*	h	[4]
3	*I. trianguliceps*	h	[16,17]
1	*I. trianguliceps*	m	[23]
2	*D. marginatus*	h	[25]
75	*D. marginatus*	l	[26]
6	*D. reticulatus*	h	HD, MM
2	*D. reticulatus*	h	[11]
4	*D. reticulatus*	h	[13]
6	*D. reticulatus*	h	[27]
10	*D. reticulatus*	m	[28]
2	*D. reticulatus*	h	[16,17]
28	*D. reticulatus*	h	[7]
242	*D. reticulatus*	h	[25]
2	*D. reticulatus*	m	[5,6]
9	*D. reticulatus*	l	[29]
2	*D. reticulatus*	l	[22]
1	*D. reticulatus*	l	[30]
3	*Ha. concinna*	h	HD, MM
1	*Ha. concinna*	h	[4]
1	*Ha. concinna*	h	[13]
3	*Ha. concinna*	l	[22]
1	*Hy. marginatum*	h	[31]
2,044	Total		

Sampling sites not referenced were contributed by the authors MM (639 sites in Lower Saxony), HD and OK (31 sites in Berlin and in the federal state of Brandenburg).

## Findings

**Figure 1:**
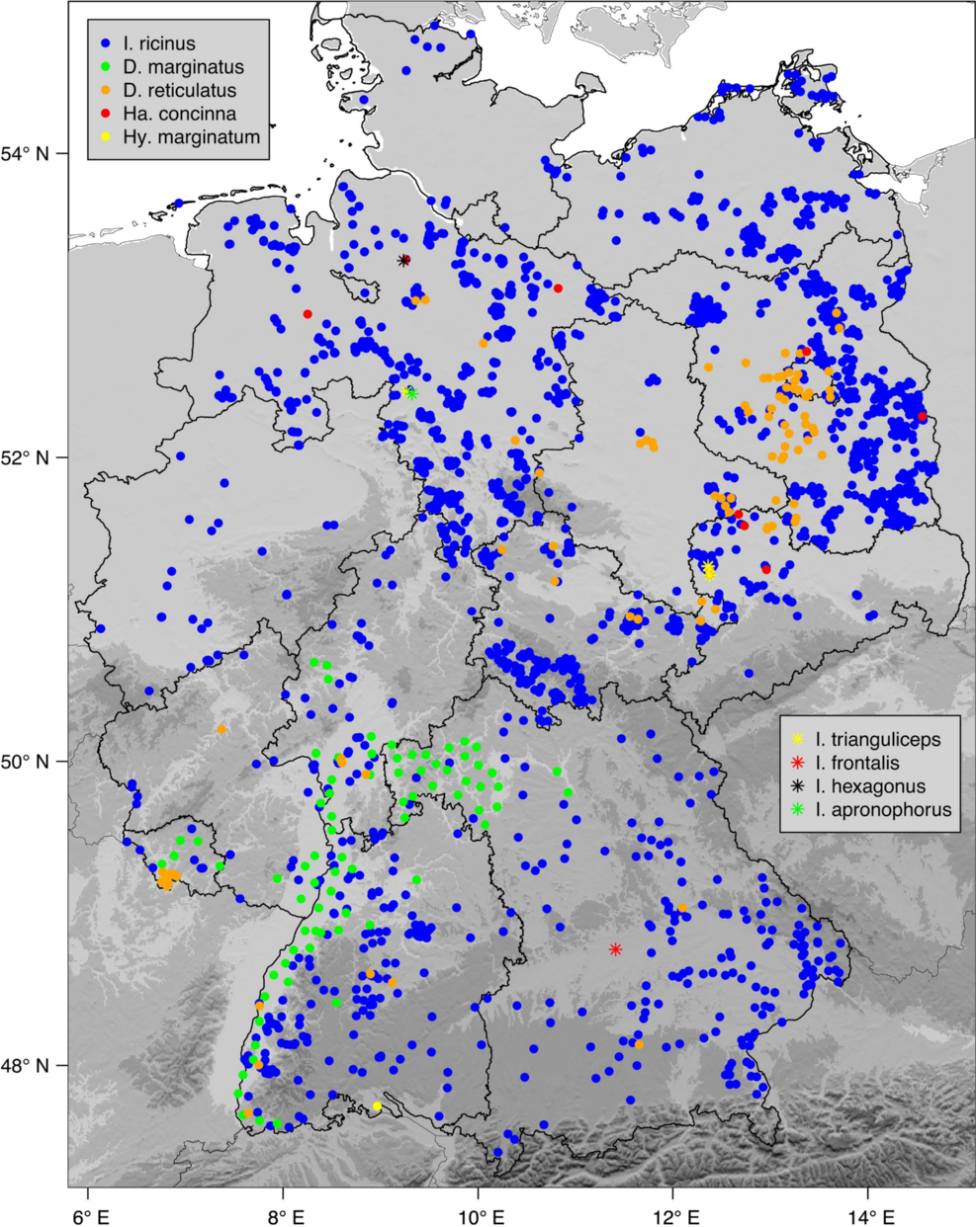
**Map of georeferenced hard tick locations in Germany.**

A total of 2,044 geographical coordinates for *Ixodes*, Dermacentor, *Haemaphysalis* and *Hyalomma* tick locations was included (Table 1) and depicted in a map (Figure 1). These coordinates include 1,855 *I. ricinus* locations, by far the most widespread and abundant ixodid tick species in Germany, occurring in the whole federal territory. A large number of georeferenced locations were taken from two already existing datasets. The first dataset, the free open access to biodiversity data collection of the *Global Biodiversity Information Facility* [9,12], lists 217 *I. ricinus* locations. The second dataset [3] comprises 776 *I. ricinus* locations. These datasets complement one another very well. Duplicates (exact matches) within and between the datasets were eliminated. Further, 661 *I. ricinus* locations were added from recent tick monitoring projects, 79 locations were extracted from the literature and 122 locations were digitized from a hand-drawn map [22]. Finally, 8 locations of rare *Ixodes* species were included, among them the first record of *I. frontalis* in Germany [24]. The number of findings of rare *Ixodes* species is low because in most tick monitoring programs they were not explicitly determined.

Two further at least regionally abundant tick species in Germany are *Dermacentor marginatus* and *Dermacentor reticulatus*. For *D. marginatus*, however, only two locations specified by geographical coordinates were provided [25]. Therefore, a historical hand-drawn map [26] was digitized to depict the well-known sources in the Rhine Valley. The latter is characterized by a warmer and dryer climate than usually found in Germany. It may be described as an extension of the Mediterranean climate to the north, a suitable habitat for the more thermophilic *D. marginatus*. The locations of *D. marginatus* were confirmed in 1990 by two of the authors, O. Kahl and H. Dautel, who found high numbers of *D. marginatus* in the region of Hammelburg (50.1° N, 9.9° O), unfortunately without documenting coordinates of the sampling sites. *D. reticulatus*, in Germany known as the marsh tick, was found in various habitats such as wastelands and meadows in many parts of Germany, except for most parts of northern Germany. It is particularly widespread and common in the federal states of Brandenburg and Berlin [7,25]. Further locations cluster in the Rhine Valley [25] and in the Saarland [28].

Additionally, 8 georeferenced locations of the relict tick *Haemaphysalis concinna* and 1 location of *Hyalomma marginatum*, the first report of an adult individual of that species in Germany [31], were included in the data collection. Further ixodid tick species listed for the fauna of Germany comprise *Ixodes arboricola*, *I. canisuga, I. lividus, I. uriae, I. vespertilionis* and *Haemaphysalis punctata* [8]. No georeferenced data were available for these species.

## Conclusions

A new map of ixodid tick distributions in Germany is presented. The map was exclusively compiled from georeferenced locations, which are provided to the scientific community via a digital dataset (see Additional file 1). The most abundant species are *I. ricinus, D. marginatus* and *D. reticulatus*. The first record of a questing adult *Hy. marginatum*, a tick frequently transported by migratory birds into Germany as a larva or nymph [32], is noteworthy [31]. This unfed female of *Hy. marginatum* was found on the leg of an ornithologist living in the vicinity of Lake Constance, a well-known resting place for migratory birds at the German-Swiss border. Note that this finding of *Hy. marginatum* in Germany is not an isolated case. A second adult individual was found in the neighbouring Switzerland [3].

The brown dog tick *Rhipicephalus sanguineus*, a species complex endemic in the Mediterranean and other warm areas worldwide, was not considered here, although it is frequently introduced to Germany by traveling dogs. Various studies on *R. sanguineus* in Germany are available. For example, 60 tick-infested dogs, 17 of which had never left Germany, were investigated [33]. The study confirmed 16 small endemic foci in private homes and animal shelters. Nevertheless, no outdoor locations of *R. sanguineus* have been documented in Germany and in other central or north European countries so far [34].

We know that there are many more non-georeferenced ixodid tick sites described in Germany in the literature and also more ixodid tick species than listed in this study [8,35]. It is an ongoing project to fill our tick map with old and new georeferenced data to make it even more useful. To document the existing knowledge about past and current tick distributions is an important basis to project current potential distributions and to model future distributions especially of prominent vector tick species.

## Additional file

Additional file 1:
**Georeferenced locations of ixodid ticks.** Digital data compiled for this study comprising geographical longitude, latitude, species, accuracy and source.
